# Chemotaxonomic Study of *Bostrychia* spp. (Ceramiales, Rhodophyta) Based on Their Mycosporine-Like Amino Acid Content

**DOI:** 10.3390/molecules25143273

**Published:** 2020-07-17

**Authors:** Maria Orfanoudaki, Anja Hartmann, Mitsunobu Kamiya, John West, Markus Ganzera

**Affiliations:** 1 Institute of Pharmacy, Pharmacognosy, University of Innsbruck, Innrain 80-82, Innsbruck 6020, Austria; maria.orfanoudaki@uibk.ac.at (M.O.); markus.ganzera@uibk.ac.at (M.G.); 2 Department of Ocean Sciences, School of Marine Resources and Environment, Tokyo University of Marine Science and Technology, Japan 4-5-7 Konan, Minato-ku, Tokyo 108-8477, Japan; mkamiy0@kaiyodai.ac.jp; 3 School of BioSciences, University of Melbourne, Parkville, Victoria 3010, Australia; jwest@unimelb.edu.au

**Keywords:** *Bostrychia* spp., *Bostrychia simpliciuscula*, classification, chemotaxonomic markers, mycosporine-like amino acids, method validation

## Abstract

This study presents a chemotaxonomic investigation of the genus *Bostrychia* through the quantitation of the major mycosporine-like amino acids (MAAs). The presence of some cryptic species had been suggested in the *B. moritziana*/*B. radicans* complex and MAA-profiling in respective samples revealed different chemotypes within this species complex. Another possibly polyphyletic species is *Bostrychia simpliciuscula*; previous molecular phylogenetic analyses showed four genetic lineages within this species, one of which was recently distinguished as a new species. Phytochemical profiling of those samples used for DNA analyses revealed four different chemotypes, corresponding to the above four lineages and it supports the re-circumscription of the other three *B. simpliciuscula* lineages. Therefore, mycosporine-like amino acids are considered as suitable chemotaxonomic markers for the reassessment of the classification of *B. simpliciuscula*. The determination of the MAA patterns in these algae was possible after developing and validating a suitable high-performance liquid chromatography–diode array detector (HPLC-DAD) method.

## 1. Introduction

Species of the genus *Bostrychia* Montagne are a prominent part of the mangrove algal flora in tropical and warm temperate environments [1]. Forty-one species are accepted taxonomically at present [2]. In 1989 King and Puttock reviewed and revised the taxonomy of this genus [3] and subsequent works have provide better insights into the physiology, mating and biogeography of the genus, leading to the reclassification of many *Bostrychia* species [4]. However, many species are still considered as polyphyletic, warranting further research for the revision of their taxonomy.

*B. simpliciuscula* is one of the species for which multiple cryptic species are known. Zuccarello et al. showed in 2018 that this species consists of four lineages that do not form a clade, but the lineages are sister to species with different morphologies. The authors showed that by branched monosiphonous laterals and the rhizoid morphology in haptera, these four lineages can be separated into two groups that, however, were still not monophyletic. According to their results, *B. simpliciuscula* is confined to the tropics, whereas one of the lineages matched a previously described species (*B. tenuissima*) found in Australasia, another lineage with the previous name *B*. *hamana-tokidae* is found in Japan, and the last lineage occurring in central New South Wales is actually a new species, *B. kingii* [5].

The *Bostrychia moritziana/B. radicans* species complex is comprised of seven molecular lineages [6], which are not reproductively inter-compatible [7]. Morphologically *B. moritziana* has been primarily distinguished by abundant compound monosiphonous lateral branches, whereas *B. radicans* had mostly polysiphonous laterals [8]. Additionally, West et al. described in 2013 a morphologically distinct lineage within the *Bostrychia moritziana*/*B. radicans* species complex as a new species, named *B. anomala* [9].

In order to perform a chemotaxonomic study on the genus *Bostrychia* an HPLC-DAD method for the quantification of MAAs in respective extracts was necessary. In the last years, several analytical studies were carried out investigating the mycosporine-like amino acid content of red algae, mainly using high-performance liquid chromatography as the preferred technique [10,11]. Yet, in most cases the number of analytes was too low to serve for a chemotaxonomic study, and therefore a HPLC-DAD method for the simultaneous determination of the twelve most common MAAs in red algae was established and validated according to the International Conference on Harmonisation (ICH) guidelines based on the determination of specificity, linearity, precision, and accuracy. Finally, the method’s applicability for the determination of MAAs in *Bostrychia* spp. was proven by showing the differentiation of species belonging to the *B*. *simpliciuscula/B. kingii* and *B. moritziana/B. radicans* complex.

## 2. Results

### 2.1. Method Development

#### 2.1.1. Sample Preparation

Prior to sample analysis optimum extraction and homogenization conditions were determined in an effort to determine the simplest and fastest method which was both reproducible and accurate. Different extraction procedures (sonication, maceration) and solvents (methanol, and mixtures of it with different water content) were investigated. Sonication-supported extraction of the plant material was found to be most efficient and 100% water the only solvent that enabled exhaustive extraction of all relevant compounds. Maceration and sonication with mixtures of water and methanol resulted either in partial extraction of the analytes or prolonged extraction time till exhaustive extraction was achieved. The sonication with higher amounts of 100% water with parallel decrease of the extraction duration or sonication repetitions (twice instead of three times) was also inadequate in terms of exhaustive extraction. Higher extraction temperatures were not investigated in order to avoid decomposition of possibly heat-sensitive analytes. The use of liquid nitrogen and a micropestle showed to be necessary for the formation of homogenously powdered plant material which was required for reproducible measurements. The sole use of the mill was sometimes impossible due to the small quantity of the available samples and even for bigger samples it was rejected because the resulting samples were not adequately homogenously milled. The efficiency of the applied extraction procedure was confirmed as follows: after preparing a sample as described in Section 4.4, the plant material was extracted once more and the supernatant analysed by HPLC. As no quantifiable amounts of the marker compounds were found in this solution, efficiency of the applied extraction procedure is proven.

#### 2.1.2. HPLC-DAD

A similar method for MAA analysis was already described in a previous work [12], however it was not validated. A further adaption of this method was necessary, because the addition of compound **6**, a main component of some *Bostrychia* spp., resulted in an overlap with compound **5**. To accomplish the simultaneous quantitation of all major compounds in the shortest separation time, required the careful assessment of all important parameters again. Similar stationary phases, different acidic modifiers, buffers and flow rates were tested as mobile phase, but this did not result in the separation of compounds **5** and **6** without a negative effect on the peak shape, selectivity and resolution of the other MAAs.

**Figure 1:**
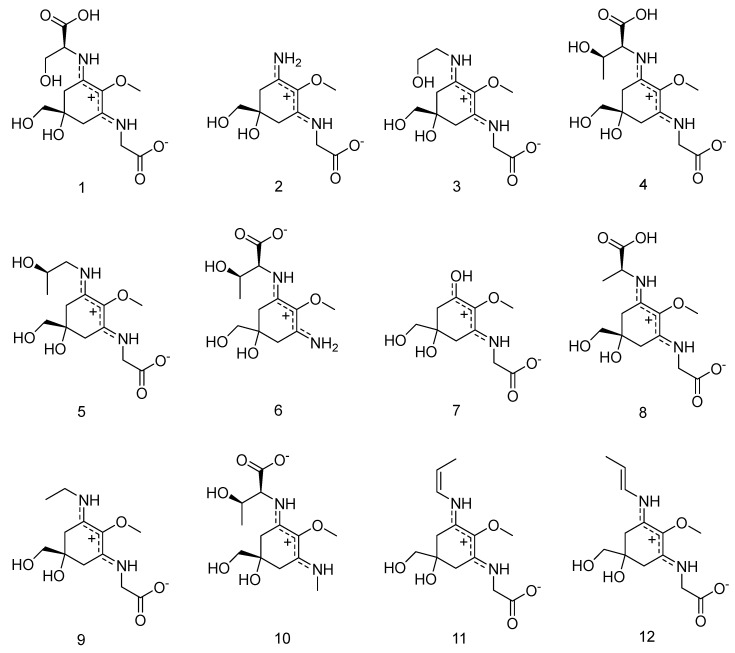
The structures of compounds **1**–**12**.

**Figure 2:**
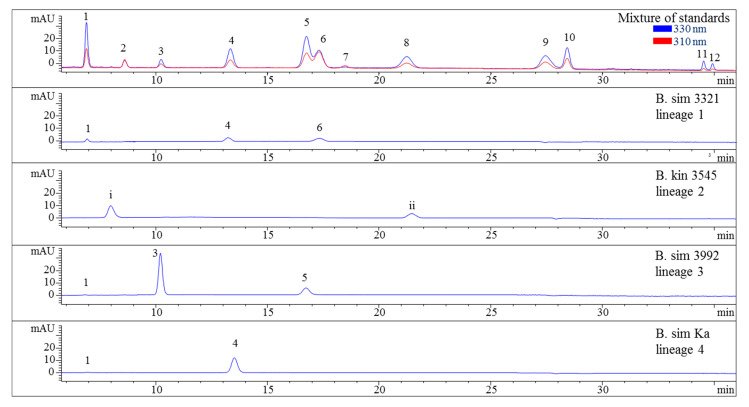
HPLC chromatograms of selected representatives of the four lineages of the *B. simpliciuscula*/*B. kingii* complex. Assignment of the compounds is according to Figure 1 and (i) unidentified MAA at 9.9 min with λmax 318 nm and *m*/*z* 294, (ii) unidentified MAA at 21.5 min with λmax 308 nm and *m*/*z* 316. The assignment of 5 as Aplysiapalythine A is tentative. Assignment of the species: B sim, *B*. *simpliciuscula*; B kin, *B*. *kingii*.

However, temperature seemed to play an important role in the separation of **5** and **6** (the lower the temperature, the better the separation), and in combination with isocratic conditions for the first twenty minutes of the separation these modifications resulted in improved separations. Now, under optimized conditions the separation of all standards (compounds **1**–**12,** Figure 1 and Figure 2) could be achieved in 40 min. Subsequently, all twelve compounds could be assigned in red algae and specifically in *Bostrychia* extracts by comparison of their retention times and UV-spectra. Assignment of compounds is as follows: shinorine (**1**), palythine (**2**), asterina-330 (**3**), porphyra-334 (**4**), aplysiapalythine A (**5**), palythine-threonine (**6**); mycosporine-glycine (**7**) mycosporine-alanine-glycine (**8**), aplysiapalythine B (**9**), mycosporine-methylamine-threonine (**10**), usujirene (**11**) and palythene (**12**). It has to be mentioned that compound **5** which was used as a standard for the method validation of this study was isolated before and structurally assigned by NMR but palythinol shows identical mass, retention time and UV spectra with it [13], therefore the assignment of compound **5** in the present study is only tentative.

### 2.2. Method Validation

Suitability of the developed HPLC method for the quantitation of compounds **1**–**12** in red algae can be deduced from several analytical parameters determined during validation. Calibration curves were constructed for compounds **1**, **4**, **5**, **8** and **10** by plotting the peak areas against standard compound concentrations, and by using a linear least square fit regression model. Calibration data presented in Table 1 indicates linearity of the method in the tested range with a determination coefficient (R^2^) higher than 0.9988 in all cases. For compounds **2**, **3**, and **8** sufficient material was not available, therefore they were quantified according to the calibration data of the structurally most similar compound **5,** whereas compound **6** was quantified according to the calibration data of compound **4**. Compounds **7**, **11** and **12** were not quantified due to stability reasons although their presence is reported in Table 2, Tables S1 and S2. Limits of detection (LOD) and limits of quantification (LOQ) ranged from 0.002 μg/mL to 0.0189 μg/mL and from 0.006 μg/mL to 0.0571 μg/mL, respectively (Table 1). Assay precision was assured by repeatedly extracting and analysing the *B. arbuscula* sample. Intraday precision was found to be lower than 3.94% and interday precision lower than 5.15% for all analytes (Table 1).

For the assurance of accuracy, five marker compounds were utilized for spiking experiments. Recovery rates of the mentioned analytes were assessed at three different concentration levels (low, medium and high) and ranged from 92.59% to 97.93% (Table 1).

### 2.3. Sample Analysis

**Figure 3:**
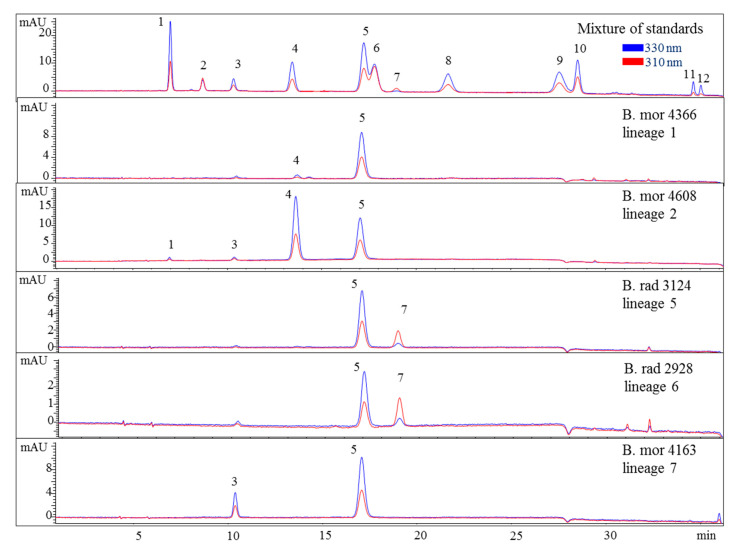
HPLC chromatograms of selected representatives of the lineages of the *B*. *moritziana*/*B*. *radicans* complex. Assignment of the compounds is according to Figure 1. The assignment of 5 as Aplysiapalythine A is tentative. Assignment of the species: B rad, *B*. *radicans*; B mor, *B*. *moritziana*.

Various samples of *Bostrychia* spp. were analysed with the developed HPLC–DAD method. Due to the number of samples investigated only a summary of the results is shown in the main manuscript (Table 2) and all detailed results can be found in the Supplementary Material (Tables S1 and S2). As can be seen in the aforementioned tables, only eight out of 12 reference compounds could be determined in these samples since none of the samples contained compounds **8**–**10** and **12**. Compounds **1** and **4** were the most frequently occurring compounds, although compound **1** was mostly present in minor concentration (lower than 0.15 mg per g of dry material). Compound **2** was only found in *B*. *arbuscula* and *B*. *tenella* samples, and compound **11** was only found in *B*. *tenella* samples. Compound **6** was the main MAA in *B*. *flagellifera* and samples of lineage 2 and 3 of *B. calliptera*, although this compound was often present in higher concentrations in the latter, which also contained compound **4** in lower amounts. *B*. *flagellifera* sometimes produced an unknown compound absorbing at 320 nm and eluting at 7.6 min in a small concentration, too. Samples of lineage 1 of the *B*. *simpliciuscula*/*B*. *kingii* complex and *B. harveyi* samples produced compounds **4** and **6** in medium (concentration between 0.15 and 1 mg per g of dry material) to high concentration (higher than 1 mg per g of dry material) and compound **1** in lower amount. Compound **3** was the main MAA only for lineage 3 of the *B. simpliciuscula/B. kingii* complex (Figure 2). Compound **5** was the only MAA found in *B. vaga* and the main MAA found in all samples of the *B. moritziana/B. radicans* complex (Figure 3).

Nevertheless, the additional presence of compound 4 enabled the differentiation of lineages 1 and 2 of the *B. moritziana/B. radicans* complex, respectively. On the other hand, samples of lineage 7 of the *B. moritziana/B. radicans* complex did not produce compound **4** but compound **3** instead. This pattern was similar to the MAA composition of *B. anomala* samples, although compound **3** was present in lower concentration. Furthermore, *B. radicans* samples (lineages 5 and 6 of the *B. moritziana/B. radicans* complex) could be distinguished from the rest of the lineages in this complex due to the presence of compound **7**. Compound **4** was the main MAA in *B. tenella*, *B. intricata*, lineage 4 of the *B. simpliciuscula/B. kingii* complex, *B. tangatensis* and *B. arbuscula*. In all of these species compound **1** was additionally present. *B. arbuscula* was the only species which produced compounds **1**–**5**, while *B. tenella* also produced compounds **6**, **7** and **11**, in addition to two unknown MAAs at 7.6 and 9.9 min. *B. intricata* and lineage 4 of the *B. simpliciuscula/B. kingii* complex only showed two MAAs, compound **1** in low and **4** in high concentration. The same compounds were also present in *B. tangatensis* and *B. radicosa* samples, but compound **4** was found in medium concentration in the first species and low concentration in the second. Finally, lineage 2 of the *B. simpliciuscula* complex, recently accepted as *B. kingii*, produced two currently unknown, new MAAs at 8 and 21.5 min, while lineage 1 of *B. calliptera* produced two unknown MAAs at 5.1 and 6.4 min.

### 2.4. LC-MS Based Analyte Identification

As shown in Table 3, HPLC-DAD-MS experiments allowed the determination of the mass of additional, unknown compounds in *Bostrychia* extracts (Figures S1–S4); their UV spectra pointed to MAAs. Due to co-elution with a high amount of non-absorbing betaines, the mass of these MAAs in *B. calliptera* at 5.1 and 6.4 min could not be identified.

## 3. Discussion

In the present work, an HPLC–DAD method was modified and validated for the determination of the major MAAs in *Bostrychia* extracts in order to separate and quantify a high number of MAAs (12 compounds), which was necessary for the discrimination of *Bostrychia* species. Moreover, satisfactory validation results for all tested parameters, including sensitivity, linearity, precision, and accuracy, and suitability for the differentiation of *Bostrychia* spp. renders the developed analytical method in general useful for examining the MAA pattern of diverse red algae, not only for *Bostrychia* species. Prior to sample analysis optimum extraction and homogenization conditions were investigated and homogenously powdered plant material was acquired only after the use of liquid nitrogen and a micropestle. The addition of methanol at the extraction solvent, the reduction of the extraction time and repetitions were deterrent for the achievement of exhaustive extraction, thus three-times sonication with pure water for 15 min was selected.

**Figure 4:**
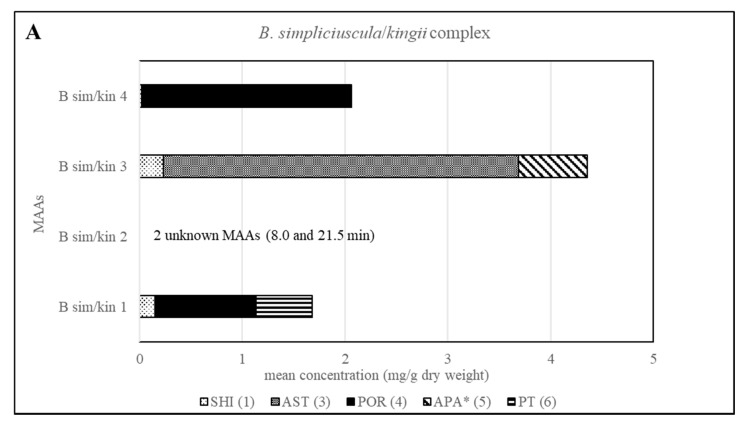
Overview of the content of MAAs expressed as mean value in mg per g of dry material for each lineage of the *B. simpliciuscula/kingii* complex. Assignment of the species: B sim, *B*. *simpliciuscula*; B kin, *B*. *kingii*.* Tentative assignment because palythinol shows identical mass, retention time and UV spectra.

The method’s suitability to differentiate the four lineages of the *B. simpliciuscula/B. kingii* complex was also evaluated and confirmed. Zuccarello et al. showed in 2018 that this species consists of four lineages with specific geographic distribution, and one of them (*B. kingii*) was accepted as a new species. The authors also reported that lineage 1 matched a previously described species, *B. tenuissima*, while lineage 4 was identical to the previously described species *B*. *hamana-tokidae*. For lineage 3 they proposed to keep the currently accepted name, *B. simpliciuscula.* Their results based on sequencing data can be confirmed by the quantitative results summarized in Table 2 and Table S1, as well as by the Figure 2 and Figure 4. Therefore, our results also support the re-circumscription of the species. Each of the four lineages has a distinct MAA pattern; lineage 1 produces both porphyra-334 and palythine-threonine as the main MAAs, while lineage 4 shows only porphyra-334 as the main MAA. Asterina-330 is the dominant MAA in lineage 3, and two unknown MAAs are present in samples of lineage 2 (*B. kingii*).

**Figure 5:**
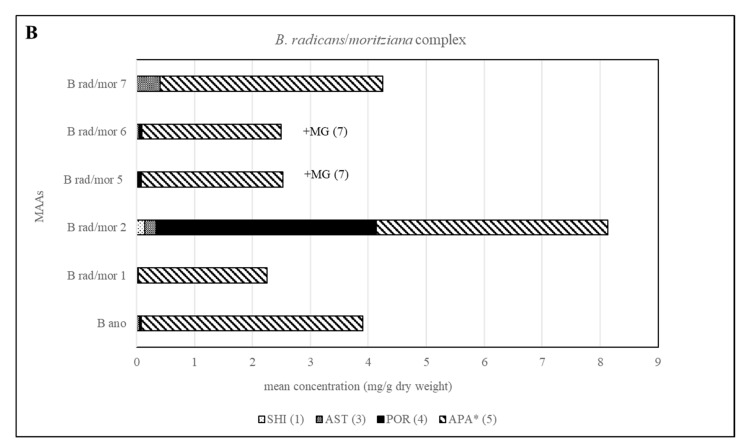
Overview of the content of MAAs expressed as mean value in mg per g of dry material for each lineage of the *B. moritziana /B*. *radicans* complex. Assignment of the species: B rad, *B*. *radicans*; B mor, *B*. *moritziana;* B ano, *B. anomala*. * Tentative assignment because palythinol shows identical mass, retention time and UV spectra.

Different chemotypes were also identified for samples belonging to the *B. moritziana/B. radicans* complex, in which seven lineages have been identified [6], and one of them was established as a new species, *B. anomala* [9]. In the present study, samples belonging to five out of the seven lineages were available, and in this case four chemotypes were identified corresponding to lineages 1, 2, 7 of this complex as well as 5 and 6, which were indistinguishable. In all cases, they contained compound **5** in amounts higher than 1 mg per g of dry material. Phytochemical investigations of these samples showed that although *B. radicans* samples belonging to lineages 5 and 6 were indistinguishable, they could be differentiated from lineages 1, 2 and 7 due to the production of mycosporine-glycine (Figure 3 and Figure 5). *B. moritziana* samples of lineage 2 produced porphyra-334 in amounts higher than 1 mg per g of dry material, while for samples of lineage 1 the respective concentration was lower. *B. moritziana* samples of lineage 7 did not produce porphyra-334 but instead a medium concentration of asterina-330 could be measured. *B. anomala* samples contained asterina-300 as well, however in lower concentration.

**Figure 6:**
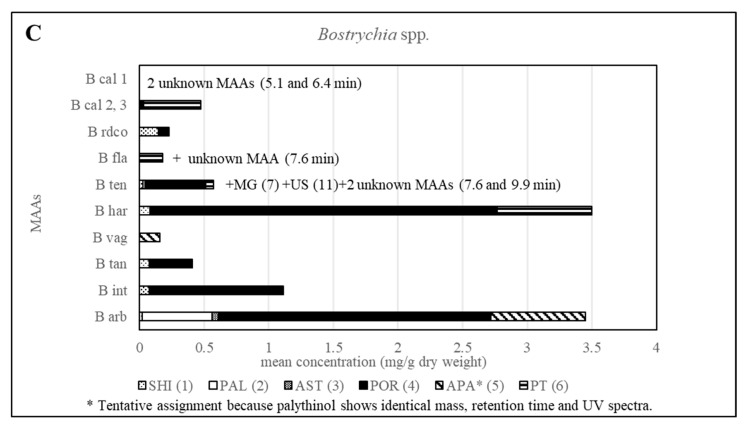
Overview of the content of MAAs expressed as mean value in mg per g of dry material for various *Bostrychia* species. Assignment of the species: B arb, *B. arbuscula*; B int, *B. intricata*; B tan, *B. tangatensis*; B vag, *B. vaga*; B har, *B. harveyi*; B ten, *B. tenella*; B fla, *B. flagellifera*; B cal, *B. calliptera*; B rdco, *B. radicosa*.

Furthermore, all other investigated *Bostrychia* spp. showed different MAA patterns, which in most cases were specific for each species (Figure 6 and Table S3), suggesting that MAAs can be considered as suitable biomarkers for this genus. Additionally, it was found that the generation, sex and developmental stage of the algae does not affect the production of MAAs since samples in different stages revealed identical MAA patterns. Besides, no consistent pattern was found in MAA quantity in relation to developmental stage or age of samples.

Finally, for each isolate of *B. tenella* and *B. flagellifera*, samples from culture and herbarium (field samples) were available. Again, regardless its origin for most samples the MAA pattern was identical and only quantitative differences could be observed for very old (in some cases more than 30 years old) samples. This was the case for MAAs with cyclohexenimine scaffold, which are known to be stable in a wide range of conditions [14]. However, mycosporine-glycine which has a cyclohexenone scaffold and is reported to be instable, was only present in culture samples. The unknown MAA at 9.9 min was also present in some *B. tenella* samples from culture but not in herbaria specimens. Surprisingly, usujirene was found in both culture and herbaria samples although it is also reported to be instable [14].

Future research perspectives of this study include the examination of other algal species, known to include cryptic species with the developed analytical method, in an effort to identify chemotaxonomic markers for them, too as well as the analysis of uninvestigated species aiming for the discovery of novel MAAs. Structure elucidation of the unknown MAAs, found in this study would be a further task requiring much higher available biomass of the algal material. Furthermore, the examination the MAA content of specific species with the developed analytical method growing under diverse environmental conditions such as various levels of salinity, nutrients supply, temperature, pH or UV radiation could reveal if the MAA production is induced under specific conditions and clarify whether MAAs serve other purposes apart from their photoprotective role such as osmoprotection or cryoprotection. Finally, re-examination of the *B. moritziana/B. radicans* complex including all seven reported lineages with a focus on different metabolites such as phenolic compounds, terpenoids or carotenoids would provide a broader overview of the chemotaxonomy of this complex and could possibly reveal markers for lineages 5 and 6 which were indistinguishable according to their MAA production.

## 4. Experimental

### 4.1. Biological Material

All of the red algae investigated were collected and morphologically identified by two of the authors (John West and Mitsunobu Kamiya) as well as by Prof. G. C. Zuccarello (Victoria University of Wellington, New Zealand), using their taxonomic expertise in conjunction with DNA sequencing data. The sequencing data are reported in previous publications which are mentioned in Tables S1 and S2. Details regarding species, collection date and place are summarized in Table S3.

### 4.2. Chemicals

Methanol for analysis had at least pro analysis (p.a.) quality and was obtained from Merck (Darmstadt, Germany). Formic acid was purchased from VWR International (Vienna, Austria) and ammonium formate from Serva (Heidelberg, Germany). Ultrapure water was produced by a Sartorius arium^®^ 611 UV (Göttingen, Germany) purification system.

### 4.3. Analyte Isolation

All standard compounds were isolated as described in [12,14]. Original NMR-spectra are available from the authors upon request. The purity of the standard compounds was assured by NMR, HPLC-DAD and HPLC-MS and was determined to be above 95%.

### 4.4. HPLC Sample Preparation for Quantitation Purposes

For the preparation of each sample, 2–40 mg plant material of *Bostrychia* spp. was frozen in liquid nitrogen and homogenized with a micropestle. This homogenization procedure was done separately for each sample used in the different experiments (quantitation purpose, assay precision, assay accuracy). For extraction, 4.0 mL of pure water was added to the algal material, mixed with a Vortex mixer (VWR) and subsequently extracted by sonication (15 min at ambient temperature). After centrifugation at 2000× *g* for 5 min the supernatant was placed in a 25 mL volumetric flask. This procedure was repeated two more times, and the flask filled up to the final volume with the extraction solvent. Afterwards, a specific volume of extract (30/divided by the mass of the dry plant material) was transferred to a vial, evaporated till dryness and redissolved in 1 mL of water 100%. This step was necessary in order to adjust the concentration so that the samples were suitable for analysis. Finally, the redissolved extract was filtered through Phenex-RC 4 mm Syringe Filters 0.45u (Phenomenex, Torrance, CA, USA).

### 4.5. General Experimental Procedures

#### 4.5.1. HPLC-DAD Analysis

HPLC analyses were performed on an Agilent 1100 series HPLC instrument, equipped with quaternary pump, autosampler, column oven, and a photodiode array detector (Agilent, Waldbronn, Germany). Optimum separation was achieved on a YMC-Pack ODS column (250 mm × 4.60 mm, 5 μm) from YMC, guarded with an in-line filter and using a mobile phase consisting of 20 mM ammonium formate and 0.25% (*v*/*v*) formic acid in water (A) and methanol (B). The applied gradient was as follows: 100% A from 0 to 20 min, 80% A at 30 min, 2% A at 35 min, and held at this composition for 5 min (total runtime of 40 min); then the column was equilibrated for 15 min under the initial conditions. Flow rate, temperature, and injection volume were adjusted to 0.65 mL/min, 7 °C, and 5.0 μL. Detection wavelengths were set to 310, 330 and 350 nm. Due to the high content of compound **4** in samples B mor 4591 T and B mor 4590 V, the injection volume had to be reduced from 5 μL to 3 μL.

#### 4.5.2. HPLC-DAD-MS Analysis

Liquid chromatography–diode array detector–electrospray ionization–mass spectrometry (LC–DAD-ESI-MS) experiments were performed on an Agilent 1260 HPLC system (Santa Clara, CA, USA), which was coupled to an amaZon iontrap mass spectrometer (Bruker, Bremen, Germany). HPLC separation conditions were identical with those described above. MS-spectra were recorded in alternating-ESI mode (capillary voltage 4.5 kV), with a drying gas temperature of 200 °C, the nebulizer gas (nitrogen) set to 4.4 psi, and a nebulizer flow (nitrogen) of 6 L/min. The scanned mass range was set between *m*/*z* 100 and 1200.

### 4.6. Calibration and Method Validation

The HPLC method used was adapted from a previously published one [11] and validated according to ICH guidelines [15] in order to ensure that it fulfilled regulatory standards. All validation results are summarized in Table 1.

#### 4.6.1. Linearity, Limit of Detection (LOD) and Limit of Quantification (LOQ)

Standard stock solutions were prepared by separately weighting and dissolving compounds **1**, **4**, **5**, **8** and **10** in water 100%. From each stock solution, at least eight calibrator levels were prepared by dilution with pure water, and each level was assayed in triplicate (see Table 1 for calibration data). Calibration curves were prepared by plotting the peak areas versus the concentrations of each analyte. The regression parameters (intercept, slope, and determination coefficient) were calculated by linear regression analysis, using Microsoft Excel. The LOD and LOQ for each analyte were calculated from the regression models, including only the lowest four dilution levels. The LOD was calculated as 3.3 times the residual standard deviation of the regression line divided by the slope of the calibration curve, whereas the LOQ was calculated as 10 time the residual standard deviation divided by the slope.

#### 4.6.2. Precision and Accuracy

Precision was assured by analysing three individually homogenized and prepared extracts of a *B. arbuscula* sample (B arb) on three consecutive days in triplicate. Intra and interday assay precision was expressed as the relative standard deviation (RSD) of the replicate quantitative measurement of compounds **1**–**5**. Accuracy was determined by recovery experiments at three different concentration levels (low, medium, high). For this purpose, individually homogenized *B. arbuscula* samples (B arb) were spiked with known amounts of analytes **1**, **4**, **5**, **8** and **10** prior to sample workup. All samples were prepared in triplicate.

## Figures and Tables

**Table 1:** Validation parameters of the HPLC method.

**Calibration Data**						
**Substance**	**Regression Equation ^a^**	**Coefficient of Determination**	**Linearity ^b^ (μg/mL)**		**LOD (μg/mL)**	**LOQ (μg/mL)**
1	y = 69632x + 2.8305	R² = 0.9997	0.0153–7.8125		0.0020	0.0060
4	y = 68147x + 2.6536	R² = 0.9998	0.0557–7.125		0.0094	0.0284
5	y = 45708x + 4.9671	R² = 0.9988	0.0669–34.250		0.0118	0.0359
8	y = 51522x + 2.3346	R² = 0.9999	0.0605–7.750		0.0101	0.0306
10	y = 32772x + 4.1515	R² = 0.9998	0.1621–41.5		0.0189	0.0571
**Accuracy and Precision**						
**Precision**			**Accuracy ^e^**			
**Substance**	**Intra-day ^c^**	**Inter-day ^d^**	**Substance**	**Low**	**Medium**	**High**
1	3.94	5.15	1	95.11 ± 1.33	92.70 ± 0.66	92.59 ± 0.32
2	3.67	3.55	4	97.93 ± 0.33	97.04 ± 0.56	97.53 ± 0.28
3	3.54	4.98	5	97.45 ± 1.57	95.78 ± 0.24	95.67 ± 0.80
4	3.76	3.67	8	97.89 ± 4.85	95.08 ± 0.64	95.29 ± 0.85
5	3.87	4.06	10	96.22 ± 4.68	95.53 ± 1.79	96.64 ± 2.22

^a^ x: concentration in mg/mL, y: AUC. ^b^ Linearity range from LLOQ to ULOQ. ^c^ Relative standard deviation within one day based on peak area in percent. ^d^ Relative standard deviation within three days based on peak area in percent. ^e^ Recovery values in percent (mean ± RSD).

**Table 2:** Overview of the MAA pattern in the investigated *Bostrychia* spp., mean values expressed as mg per g of dry material and standard deviation is reported in parenthesis. (detailed results can be found in supplementary material, Tables S1 and S2), assignment of compounds: SHI, Shinorine (**1**); PAL, Palythine (**2**); AST, Asterina-330 (**3**); POR, Porphyra-334 (**4**); APA, Aplysiapalythine A (**5**); PT, Palythine-Threonine (**6**); MG, Mycosporine-Glycine (**7**) and US, Usujirene (**11**). Assignment of the species: B sim, *B*. *simpliciuscula*; B kin, *B*. *kingii*; B rad, *B*. *radicans*; B ano, *B*. *anomala*; B mor, *B*. *moritziana*; B arb, *B*. *arbuscula*; B int, *B*. *intricata*; B tan, *B*. *tangatensis*; B vag, *B*. *vaga*; B har, *B*. *harveyi*; B ten, *B*. *tenella*; B fla, *B*. *flagellifera*; B cal, *B*. *calliptera*; B rdco, *B*. *radicosa*.

Sample	SHI (1)	PAL (2)	AST (3)	POR (4)	APA* (5)	PT (6)	MG (7)	US (11)	MAA at 7.6 min	MAA at 9.9 min	MAA at 8.0 min	MAA at 21.5 min	MAAs at 5.1 and 6.4 min
B cal 1													Present
B cal 2,3				0.03 (±0.05)		0.44 (±0.41)							
B fla						0.18 (±0.22)			Sometimes Present				
B har	0.08			2.689		0.731							
B ten	0.03 (±0.12)	0.01 (±0.08)	0.01 (±0.08)	0.46 (±0.46)		0.06 (±0.07)	Sometimes Present	Sometimes Present	Present	Sometimes Present			
B sim 1	0.15 (±0.08)			0.98 (±0.49)		0.55 (±0.29)							
B sim 4	0.02 (±0.01)			2.04 (±1.07)									
B int	0.07 (±0.08)			1.04 (±0.26)									
B tan	0.07 (±0.04)			0.34 (±0.11)									
B rdco	0.15 (±0.21)			0.08 (±0.02)									
B arb	0.01	0.54	0.05	2.11	0.74								
B ano			0.37 (±0.42)	0.11 (±0.22)	3.46 (±1.15)								
B rad/mor 1				0.03 (±0.04)	2.22 (±0.88)								
B rad/mor 2	0.14 (±0.18)		0.19 (±0.14)	3.80 (±2.34)	4.00 (±1.49)								
B rad/mor 5			0.004 (±0.01)	0.08 (±0.15)	2.44 (±1.35)		Present						
B rad/mor 6			0.04 (±0.08)	0.05 (±0.1)	2.40 (±0.87)		Present						
B rad/mor 7			0.41 (±0.39)		3.84 (±0.46)								
B vag					0.15								
B sim 3	0.23 (±0.23)		3.45 (±2.95)		0.67 (±0.95)								
B kin (B sim 2)											Present	Present	

* Tentative assignment because palythinol shows identical mass, retention time and UV spectra.

**Table 3:** Absorption maxima and molecular mass of unidentified compounds, most likely MAAs, in the extracts of *Bostrychia* spp.

Retention Time	λmax (nm)	Mass (Da)
7.6	320	274
8.0	334	403
9.9	318	294
21.5	308	316

## Supplementary Materials

The following are available online. UV and mass spectra of the unidentified MAAs, collection sites and dates of the investigated samples of *Bostrychia* spp. and detailed quantitative HPLC-DAD results for compounds **1**–**12** in each *Bostrychia* spp.
