# Supplementary material for: Chemotaxonomic Study of Bostrychia spp. (Ceramiales, Rhodophyta) Based on Their Mycosporine-Like Amino Acid Content
Source: Molecules. 2020 Jul 17;25(14):3273. doi: 10.3390/molecules25143273 (PMC7397226; doi:10.3390/molecules25143273)
Supplement: Supplementary file 1 [file molecules-25-03273-s001.pdf]

molecules

Supplementary Material

Chemotaxonomic Study of *Bostrychia* spp. (Ceramiales, Rhodophyta) Based on Their Mycosporine-like Amino Acid Content

Maria Orfanoudaki [1], Anja Hartmann [1,*], Mitsunobu Kamiya [2], John West [3] and Markus Ganzera [1]

[1] Institute of Pharmacy, Pharmacognosy, University of Innsbruck, Innrain 80-82, Innsbruck 6020, Austria; maria.orfanoudaki@uibk.ac.at (M.O.); markus.ganzera@uibk.ac.at (M.G.)
[2] Department of Ocean Sciences, School of Marine Resources and Environment, Tokyo University of Marine Science and Technology, Japan 4-5-7 Konan, Minato-ku, Tokyo 108-8477, Japan; mkamiy0@kaiyodai.ac.jp (M. K.)
[3] School of BioSciences, University of Melbourne, Parkville, 3010 Victoria, Australia; jwest@unimelb.edu.au (J. W.)
* Correspondence: anja.hartmann@uibk.ac.at (A.H.); Tel.: +43 512 507-58430

Contents

Figure S1 UV and Mass spectrum of compound the unidentified MAA at 7.6 min 2

Figure S2 UV and Mass spectrum of compound the unidentified MAA at 9.9 min 2

Figure S3 UV and Mass spectrum of compound the unidentified MAA at 8.0 min 3

Figure S4 UV and Mass spectrum of compound the unidentified MAA at 21.2 min 4

Table S1. Quantitative HPLC-DAD results for compounds **1**-**12** in the *B. simpliciuscula*/*B. kingii* and the *B. moritziana*/*B. radicans* complex...5

Table S2. Quantitative HPLC-DAD results for compounds **1**-**12** in *Bostrychia* spp. 10

Table S3. Overview of the investigated samples of *Bostrychia* spp., their collection sites and dates...16

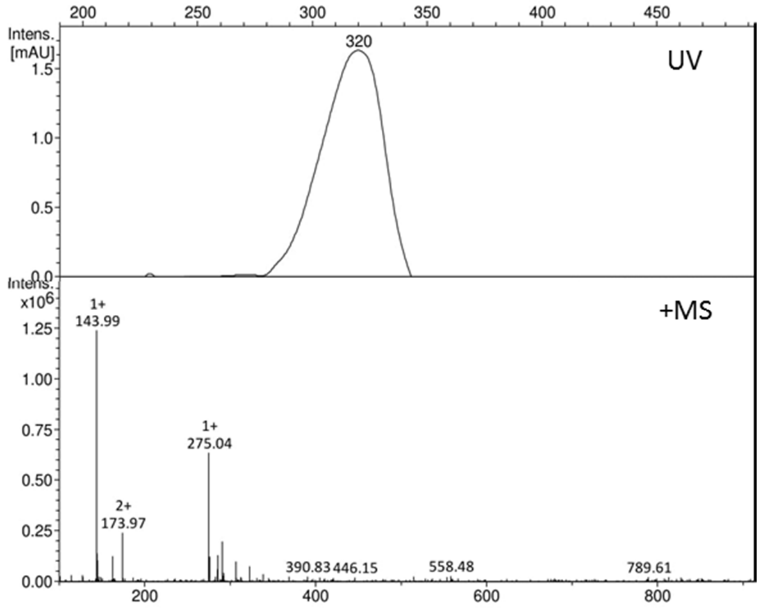

Figure S1. UV and mass spectrum of compound the unidentified MAA at 7.6 min

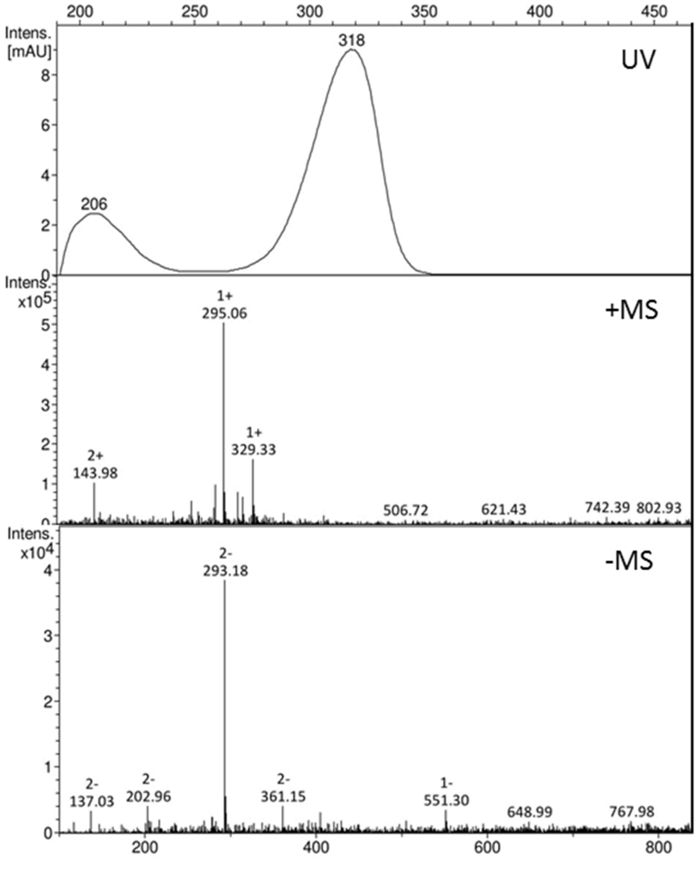

Figure S2. UV and mass spectrum of compound the unidentified MAA at 9.9 min

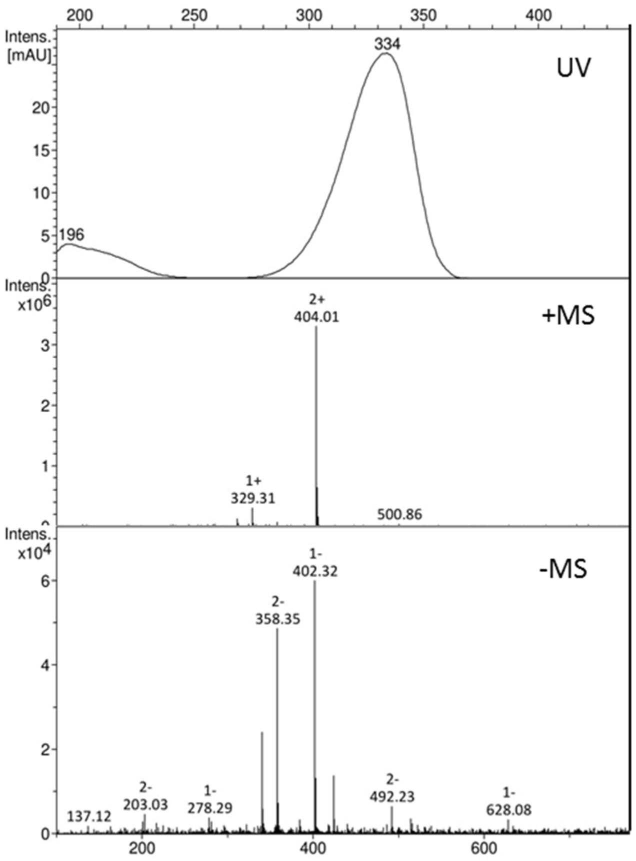

Figure S3. UV and mass spectrum of compound the unidentified MAA at 8.0 min

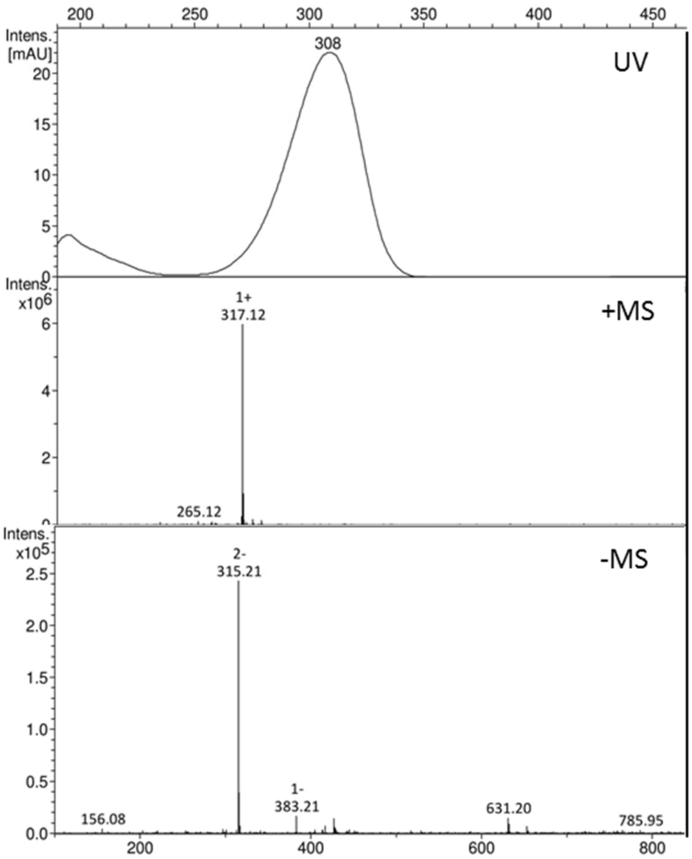

Figure S4. UV and mass spectrum of compound the unidentified MAA at 21.2 min

Table S1. Quantitative HPLC-DAD results for compounds 1-12 in the *B. simpliciuscula*/*B. kingii* and the *B. moritziana*/*B. radicans* complex.; all values expressed as mg per g of dry material, assignment of compounds and species is the same as Table 2, assignment of generation, sex and developmental stage: B, Bisexual; F, Female; M, Male; T, Tetrasporophyte; G, Gametophyte; S, Sporelings; V, Vegetative, assignment of sample origin: H, Herbarium; C, Culture.

Sample	SHI (**1**)	AST (**3**)	POR (**4**)	APA* (**5**)	PT (**6**)	MG (**7**)	Unknown MAA	Lineage [1,2]	Developmental stage	Sample origin
**B sim C 3306 T**	0.058		1.358		1.570			B sim/kin 1	Tetrasporophyte	Culture
**B sim C 3321 T**	0.191		0.686		0.783			B sim/kin 1	Tetrasporophyte	Culture
**B sim C 3677 T**	0.196		0.940		0.379			B sim/kin 1	Tetrasporophyte	Culture
**B sim C 3322 F**	0.139		0.907		0.473			B sim/kin 1	Female	Culture
**B sim C 3305**	0.190		2.182		0.974			B sim/kin 1		Culture
**B sim C 3671 T**	0.132		0.691		0.406			B sim/kin 1	Tetrasporophyte	Culture
**B sim C 3663 MF**			0.328		0.302			B sim/kin 1	Male, Female	Culture
**B sim C 3663 G**	0.144		1.439		0.233			B sim/kin 1	Gametophyte	Culture
**B sim C 3663 F**	0.101		1.628		0.651			B sim/kin 1	Female	Culture
**B sim C 3663 T**	0.060		0.519		0.31			B sim/kin 1	Tetrasporophyte	Culture
**B sim C 3581 T**	0.142		0.641		0.472			B sim/kin 1	Tetrasporophyte	Culture
**B sim C 3581 S**	0.321		1.989		0.563			B sim/kin 1	Sporelings	Culture
**B sim C 3581 F**	0.076		0.835		0.517			B sim/kin 1	Female	Culture
**B sim C 3304 T**	0.148		0.879		0.467			B sim/kin 1	Tetrasporophyte	Culture
**B sim C 3108 T**	0.085		0.429		0.291			B sim/kin 1	Tetrasporophyte	Culture
**B sim C 3108 M**	0.126		1.126		0.338			B sim/kin 1	Male	Culture
**B sim C 3108 F**	0.320		0.892		0.222			B sim/kin 1	Female	Culture
**B sim C 3658 T**	0.085		0.522		0.413			B sim/kin 1	Tetrasporophyte	Culture
**B sim C 3658 G**	0.129		1.746		0.438			B sim/kin 1	Gametophyte	Culture
**B sim C 3658 F**	0.160		1.493		0.382			B sim/kin 1	Female	Culture

Table S1. *Cont.*

**B sim C 3576**	0.176		0.492		0.428			B sim/kin 1		Culture
**B sim C 3319 T**	0.257		1.494		0.72			B sim/kin 1	Tetrasporophyte	Culture
**B sim C 2915 G**	0.079		0.310		0.196			B sim/kin 1	Gametophyte	Culture
**B sim C 2915 T**	0.094		0.665		0.787			B sim/kin 1	Tetrasporophyte	Culture
**B sim C 2915 S**	0.201		0.527		0.233			B sim/kin 1	Sporelings	Culture
**B sim C 2747**	0.240		1.408		0.656			B sim/kin 1		Culture
**B sim C 3315 T**	0.120		0.713		0.955			B sim/kin 1	Tetrasporophyte	Culture
**B sim C 3672**	0.158		0.676		0.484			B sim/kin 1		Culture
**B sim C 3657 T**	0.033		0.506		0.512			B sim/kin 1	Tetrasporophyte	Culture
**B sim C 3612 T**	0.170		1.638		0.264			B sim/kin 1	Tetrasporophyte	Culture
**B sim C 3612 B**	0.191		1.256		0.668			B sim/kin 1	Bisexual	Culture
**B sim C 3612 F**	0.126		0.801		0.516			B sim/kin 1	Female	Culture
**B sim C 3317 T**	0.276		1.202		0.881			B sim/kin 1	Tetrasporophyte	Culture
**B sim C 4046**	0.270		0.447		0.659			B sim/kin 1		Culture
**B sim C 2933**	0.124		0.987		1.120			B sim/kin 1		Culture
**B kin C 4203 F**							At 8.0 and 21.5 min	B sim/kin 2	Female	Culture
**B kin C 3545**							At 8.0 and 21.5 min	B sim/kin 2		Culture
**B kin C 3109 F**							At 8.0 and 21.5 min	B sim/kin 2	Female	Culture
**B kin C 3110 F**							At 8.0 and 21.5 min	B sim/kin 2	Female	Culture
**B kin C 3111 F**							At 8.0 and 21.5 min	B sim/kin 2	Female	Culture
**B kin C 3114 F**							At 8.0 min	B sim/kin 2	Female	Culture
**B kin C 3114 T**							At 8.0 min	B sim/kin 2	Tetrasporophyte	Culture
**B kin C 3114 MT**							At 8.0 min	B sim/kin 2	Male, Tetrasporophyte	Culture

Table S1. *Cont.*

**B kin C 3308 TF**							At 8.0 min	B sim/kin 2	Tetrasporophyte, Female	Culture
**B kin C 3308 F**							At 8.0 min	B sim/kin 2	Female	Culture
**B sim C 2964 M**	0.347	1.914						B sim/kin 3	Male	Culture
**B sim C 3211 T**		0.536		0.24				B sim/kin 3	Tetrasporophyte	Culture
**B sim C 2963 B**	0.316	1.879						B sim/kin 3	Bisexual	Culture
**B sim C 2963 T**	0.597	2.051						B sim/kin 3	Tetrasporophyte	Culture
**B sim C 3992**	0.044	7.711		1.927				B sim/kin 3		Culture
**B sim C 3546**	0.099	6.627		1.857				B sim/kin 3		Culture
**B sim H Oo**	0.013		1.287					B sim/kin 4		Herbarium
**B sim H Ka**	0.033		2.797					B sim/kin 4		Herbarium
**Sample**	SHI (**1**)	AST (**3**)	POR (**4**)	APA* (**5**)	PT (**6**)	MG (**7**)	Unknown MAA	Lineage [3–7]		
**B rad C 3029 F**				2.415		Present		B rad/mor 5	Female	Culture
**B rad C 3038 T**				2.781		Present		B rad/mor 5	Tetrasporophyte	Culture
**B rad C 3034 F**				0.898		Present		B rad/mor 5	Female	Culture
**B rad C 3061 MF**				2.114		Present		B rad/mor 6	Male, Female	Culture
**B rad C 2928 F**				1.368		Present		B rad/mor 6	Female	Culture
**B rad C 3129 MF**				1.900		Present		B rad/mor 5	Male, Female	Culture
**B rad C 3136 T**				2.165		Present		B rad/mor 5	Tetrasporophyte	Culture
**B rad C 3124 MF**				2.011		Present		B rad/mor 5	Male, Female	Culture
**B rad C 2649**				2.731		Present		B rad/mor 6		Culture
**B rad C 2636**				0.793		Present				Culture
**B rad C 3030 F**				1.632		Present		B rad/mor 5	Female	Culture
**B rad C 3128 F**				0.193		Present		B rad/mor 5	Female	Culture

Table S1. *Cont.*

**B rad C 3171 T**				0.913		Present		B rad/mor 5		Culture
**B rad C 3137**				1.446		Present				Culture
**B rad C 3044 M**				0.211		Present			Male	Culture
**B rad C 3195 T**				3.082		Present		B rad/mor 5	Tetrasporophyte	Culture
**B rad C 3115 T**			0.030	2.503		Present		B rad/mor 5	Tetrasporophyte	Culture
**B rad C 3037 T**			0.151	2.446		Present		B rad/mor 5	Tetrasporophyte	Culture
**B rad C 3026 MF**			0.337	2.858		Present		B rad/mor 5	Male, Female	Culture
**B rad C 3062 T**			0.213	3.590		Present			Tetrasporophyte	Culture
**B rad C 3035 F**			0.040	2.287		Present		B rad/mor 5	Female	Culture
**B rad C 3051 T**			0.084	2.827		Present		B rad/mor 5	Tetrasporophyte	Culture
**B rad C 3058 MF**			0.027	1.933		Present			Male, Female	Culture
**B rad C 3144 MF**			0.040	1.670		Present			Male, Female	Culture
**B rad C 3125 MF**		0.042	0.172	4.319		Present			Male, Female	Culture
**B rad C 3094 F**		0.156	0.200	3.406				B rad/mor 6	Female	Culture
**B rad C 3203 V**		0.346	0.310	4.396		Present			Vegetative	Culture
**B rad C 3142 T**		0.029	0.558	4.476		Present		B rad/mor 5	Tetrasporophyte	Culture
**B rad C 3126 F**	0.031	0.033	0.100	4.857		Present			Female	Culture
**B rad C 3140 M**		0.130	0.305	5.831		Present			Male	Culture
**B rad C 3019 F**		0.044	0.162	6.080		Present		B rad/mor 5	Female	Culture
**Sample**	SHI (**1**)	AST (**3**)	POR (**4**)	APA* (**5**)	PT (**6**)	MG (**7**)	Unknown MAA	Lineage [3–8]		
**B mor C 4170**				3.330				B rad/mor 1		Culture
**B mor C 4169**				1.846				B rad/mor 1		Culture
**B mor C 4168**				1.422				B rad/mor 1		Culture
**B mor C 4167**				0.826				B rad/mor 1		Culture

Table S1. *Cont.*

**B mor C 3905 M**				2.032				B rad/mor 1	Male	Culture
**B mor C 3799**				2.816				B rad/mor 1		Culture
**B mor C 3700**				1.129				B rad/mor 1		Culture
**B mor C 3699**				1.212				B rad/mor 1		Culture
**B mor C 3659 T**				2.524				B rad/mor 1	Tetrasporophyte	Culture
**B mor C 4383 T**				1.340				B rad/mor 1	Tetrasporophyte	Culture
**B mor C 4371 T**			0.030	2.340				B rad/mor 1	Tetrasporophyte	Culture
**B mor C 4369 T**			0.041	3.497				B rad/mor 1	Tetrasporophyte	Culture
**B mor C 4164**			0.031	2.531				B rad/mor 1		Culture
**B mor C 4366 FT**			0.121	3.662				B rad/mor 1	Female, Tetrasporophyte	Culture
**B mor C 4309 T**			0.087	2.833				B rad/mor 1	Tetrasporophyte	Culture
**B mor C 4310 T**			0.127	2.204				B rad/mor 1	Tetrasporophyte	Culture
**B mor C 4278 V**		0.207		3.330				B rad/mor 7	Vegetative	Culture
**B mor C 4163**		0.927		4.061				B rad/mor 7		Culture
**B mor C 4160**		0.459		4.377				B rad/mor 7		Culture
**B mor C 4631**		0.032		3.607				B rad/mor 7		Culture
**B mor C 4367 T**		0.047	0.442	1.797				B rad/mor 2	Tetrasporophyte	Culture
**B mor C 4609 T**		0.092	1.404	4.717				B rad/mor 2	Tetrasporophyte	Culture
**B mor C 4592 T**	0.014		1.417	1.297					Tetrasporophyte	Culture
**B mor C 4591 T**	0.139	0.090	6.250	3.932				B rad/mor 2	Tetrasporophyte	Culture
**B mor C 4608**	0.086	0.168	4.267	4.848				B rad/mor 2		Culture
**B mor C 4633**	0.029	0.104	1.702	2.819				B rad/mor 2		Culture
**B mor C 4590 V**	0.598	0.203	7.339	1.997				B rad/mor 2	Vegetative	Culture

Table S1. *Cont.*

**B mor C 3001 T**	0.201	0.492	4.091	5.314				B rad/mor 2	Tetrasporophyte	Culture
**B mor C 4596 F**	0.149	0.214	5.562	4.464				B rad/mor 2	Female	Culture
**B mor C 4368 T**	0.079	0.322	3.145	6.080				B rad/mor 2	Tetrasporophyte	Culture
**Sample**	SHI (1)	AST (3)	POR (4)	APA* (5)	PT (6)	MG (7)	Unknown MAA	[9]		
**B ano C 4613 TMF**		0.065		3.084					Tetrasporophyte, Male, Female	Culture
**B ano C 4588 V**		0.038	0.059	4.571					Vegetative	Culture

* Tentative assignment because palythinol shows identical mass, retention time and UV spectra.

Table S2. Quantitative HPLC-DAD results for compounds 1-12 in *Bostrychia* spp.; all values expressed as mg per g of dry material, assignment of compounds and species is the same as Table 2, assignment of generation, sex, developmental stage and sample origin is the same as Table S1.

**Sample**	**SHI (1)**	**PAL (2)**	**AST (3)**	**POR (4)**	**APA* (5)**	**PT (6)**	**MG (7)**	**US (11)**	**Unknown MAA**	**[10]**	**Developmental stage**	**Sample origin**
**B arb H**	0.018	0.541	0.052	2.105	0.735							Herbarium
**Sample**	SHI (1)	PAL (2)	AST (3)	POR (4)	APA* (5)	PT (6)	MG (7)	US (11)	Unknown MAA	[11]		
**B int C 2952**	0.044			1.250								Culture
**B int C 2951 T**	0.011			0.752							Tetrasporophyte	Culture
**B int C 4557**	0.163			1.124								Culture
**Sample**	SHI (1)	PAL (2)	AST (3)	POR (4)	APA* (5)	PT (6)	MG (7)	US (11)	Unknown MAA	[12,13]		
**B tan C 3242 MFT**	0.091			0.385						Tetrasporophyte	Tetrasporophyte, Male, Female	Culture
**B tan C 3241 MFT**	0.016			0.186							Tetrasporophyte, Male, Female	Culture
**B tan C 3240 MFT**	0.117			0.468							Tetrasporophyte, Male, Female	Culture

Table S2. *Cont.*

**B tan C 3239 MFT**	0.053			0.260							Tetrasporophyte, Male, Female	Culture
**B tan C 3238 MFT**	0.082			0.392							Tetrasporophyte, Male, Female	Culture
**Sample**	SHI (**1**)	PAL (**2**)	AST (**3**)	POR (**4**)	APA* (**5**)	PT (**6**)	MG (**7**)	US (**11**)	Unknown MAA	[11]		
**B vag C 3781**					0.154							Culture
**Sample**	SHI (**1**)	PAL (**2**)	AST (**3**)	POR (**4**)	APA* (**5**)	PT (**6**)	MG (**7**)	US (**11**)	Unknown MAA	[14]		
**B har C 3908 F**	0.080			2.689		0.731					Female	Culture
**Sample**	SHI (**1**)	PAL (**2**)	AST (**3**)	POR (**4**)	APA* (**5**)	PT (**6**)	MG (**7**)	US (**11**)	Unknown MAA	[15]		
**B ten H 4311 T**				0.296		0.098			At 7.6 min		Tetrasporophyte	Herbarium
**B ten C 2985**												Culture
**B ten H 2985 F**				0.174					At 7.6 min		Female	Herbarium
**B ten C 3706**									At 9.9 min			Culture
**B ten C 4528 V**				0.055					At 7.6 min		Vegetative	Culture
**B ten H 2765 T**				0.205					At 7.6 min		Tetrasporophyte	Herbarium
**B ten C 2515**				0.412		0.158			At 7.6 min			Culture
**B ten C 4552 V**	0.012			0.252		0.051	Present		At 7.6 min		Vegetative	Culture
**B ten H 4552 V**				0.206		0.047			At 7.6 min		Vegetative	Herbarium
**B ten C 2722**				0.140		0.042			At 7.6 min			Culture
**B ten H 2722**	0.033			0.356		0.046			At 7.6 min			Herbarium
**B ten C 2850**				0.167		0.054			At 7.6 and 9.9 min			Culture
**B ten H 2850 T**				0.052					At 7.6 and 9.9 min		Tetrasporophyte	Herbarium

Table S2. *Cont.*

**B ten C 4229 M**	0.021			0.466					At 7.6 and 9.9 min		Male	Culture
**B ten H 4229 M**				0.643					At 7.6 min		Male	Herbarium
**B ten C 4089 B**	0.033	0.036		0.863				Present	At 7.6 min		Bisexual	Culture
**B ten H 4089 B**				0.091					At 7.6 min		Bisexual	Herbarium
**B ten C 3727**				0.045		<LOQ			At 7.6 min			Culture
**B ten H 3727 M**				0.075					At 7.6 min		Male	Herbarium
**B ten C 2998 T**				0.396		0.105	Present	Present	At 7.6 min		Tetrasporophyte	Culture
**B ten H 2998 T**	0.012			1.241		0.296			At 7.6 min		Tetrasporophyte	Herbarium
**B ten C 2908**				0.084			Present		At 7.6 and 9.9 min			Culture
**B ten H 2908**				0.296					At 7.6 min			Herbarium
**B ten C 2905**				0.090					At 7.6 min			Culture
**B ten H 2905 T**	0.099			1.900		0.097			At 7.6 min		Tetrasporophyte	Herbarium
**B ten C 2815**				0.035			Present		At 7.6 min			Culture
**B ten H 2815 T**				0.499		0.118			At 7.6 min		Tetrasporophyte	Herbarium
**B ten C 4662**				0.377		0.136		Present	At 7.6 min			Culture
**B ten H 4662 V**				0.390		0.101		Present	At 7.6 min		Vegetative	Herbarium
**B ten C 3807**	0.788			0.909					At 7.6 and 9.9 min			Culture
**B ten H 3807**				0.681		0.035			At 7.6 min			Herbarium
**B ten C 3079**				0.235		0.034			At 7.6 and 9.9 min			Culture
**B ten H 3079**	0.467	0.572	0.643	2.334					At 7.6 min			Herbarium
**B ten C 2767**				0.339		0.105			At 7.6 and 9.9 min			Culture

Table S2. *Cont.*

**B ten H 2767 F**				0.818		0.224			At 7.6 min		Female	Herbarium
**B ten C 4116 F**				0.330		0.097			At 7.6 min		Female	Culture
**B ten H 4116 F**				0.470		0.14			At 7.6 min		Female	Herbarium
**B ten C 2979 T**				0.097					At 7.6 min		Tetrasporophyte	Culture
**B ten H 2979 T**				0.542		0.057			At 7.6 min		Tetrasporophyte	Herbarium
**B ten C 2978**	0.013			0.295		0.037	Present		At 7.6 min			Culture
**B ten H 2978**				1.014		0.164			At 7.6 min			Herbarium
**B ten C 2764**				0.397		0.056			At 7.6 min			Culture
**B ten H 2764 F**				0.264		0.043		Present	At 7.6 min		Female	Herbarium
**B ten C 2904**				0.299		0.054			At 7.6 min			Culture
**B ten H 2904 T**				0.934		0.255			At 7.6 min		Tetrasporophyte	Herbarium
**B ten C 4081 F**				1.397		0.159			At 7.6 min		Female	Culture
**B ten H 4081 F**				0.809		0.088			At 7.6 min		Female	Herbarium
**B ten C 4081 M**				0.695		0.065			At 7.6 min		Male	Culture
**B ten H 4081 M**				0.403				Present	At 7.6 min		Male	Herbarium
**B ten C 3417**				0.216		0.047	Present		At 7.6 min			Culture
**B ten H 3417 F**	0.026	0.078		1.201		0.165			At 7.6 min		Female	Herbarium
**B ten C 4434 V**				0.390		0.043		Present	At 7.6 min		Vegetative	Culture
**B ten H 4434 V**				0.031					At 7.6 min		Vegetative	Herbarium
**B ten C 2751**				0.409		0.074			At 7.6 min			Culture
**B ten H 2751 F**	0.015	0.124		0.497		0.096			At 7.6 min		Female	Herbarium
**B ten C 4096 F**	0.052			0.839					At 7.6 min		Female	Culture
**B ten H 4096 F**				0.223					At 7.6 min		Female	Herbarium
**B ten C 3635**				0.307					At 7.6 min			Culture

Table S2. *Cont.*

**B ten H 3635 T**				0.111					At 7.6 min		Tetrasporophyte	Herbarium
**Sample**	SHI (**1**)	PAL (**2**)	AST (**3**)	POR (**4**)	APA* (**5**)	PT (**6**)	MG (**7**)	US (**11**)	Unknown MAA	[14,16]		
**B fla C 4400 T**						0.08					Tetrasporophyte	Culture
**B fla H 4400**						0.111						Herbarium
**B fla C 3553 T**						0.067					Tetrasporophyte	Culture
**B fla H 3553**						0.093						Herbarium
**B fla H 3821 T**						0.043					Tetrasporophyte	Herbarium
**B fla C 3821 T**						0.047					Tetrasporophyte	Culture
**B fla C 3884 T**						0.063					Tetrasporophyte	Culture
**B fla H 3884 T**						0.051					Tetrasporophyte	Herbarium
**B fla C 4218 T**						0.303			At 7.6 min		Tetrasporophyte	Culture
**B fla H 4218 T**						0.802			At 7.6 min		Tetrasporophyte	Herbarium
**B fla C 4230**						0.049						Culture
**B fla H 4230**						0.236			At 7.6 min			Herbarium
**B fla H 3113**						0.396			At 7.6 min			Herbarium
**Sample**	SHI (**1**)	PAL (**2**)	AST (**3**)	POR (**4**)	APA* (**5**)	PT (**6**)	MG (**7**)	US (**11**)	Unknown MAA	Lineage [16,17]		
**B cal C 4097**						0.479				B cal 2		Culture
**B cal C 3190 T**				0.118		0.881				B cal 3	Tetrasporophyte	Culture
**B cal C 3066 T**				0.053		0.579				B cal 3	Tetrasporophyte	Culture
**B cal C 3194 MF**				0.089		0.683				B cal 3	Male, Female	Culture
**B cal C 3042**						1.036				B cal 3		Culture
**B cal C 3041 T**				0.072		0.754				B cal 3	Tetrasporophyte	Culture

Table S2. *Cont.*

**B cal C 3414**									At 5.1 and 6.4 min	B cal 1		Culture
**B cal C 3054 T**									At 5.1 and 6.4 min	B cal 1	Tetrasporophyte	Culture
**B cal C 3016**									At 5.1 and 6.4 min	B cal 1		Culture
**B cal C 3216 M**									At 5.1 and 6.4 min	B cal 1	Male	Culture
**Sample**	SHI (**1**)	PAL (**2**)	AST (**3**)	POR (**4**)	APA* (**5**)	PT (**6**)	MG (**7**)	US (**11**)	Unknown MAA	[14]		
**B rdco C 4271**				0.058								Culture
**B rdco C 4178**				0.095								Culture
**B rdco C 4086**	0.131			0.067								Culture
**B rdco C 3744**	0.452			0.099								Culture

* Tentative assignment because palythinol shows identical mass, retention time and UV spectra.

Table S3. Overview of the investigated samples of *Bostrychia* spp., their collection sites and dates.

Sample	Collection place	Collection Date
B sim C 3306 T	Batemans Bay, NSW, AUS	01/12/1993
B sim C 3321 T	Wapengo Lake, NSW AUS	01/12/1993
B sim C 3677 T	Rhyll, Phillip Island, VIC, AUS	27/12/1996
B sim C 3322 F	Merimbula, NSW, AUS	01/12/1993
B sim C 3305	Bermagui R., NSW, AUS	01/12/1993
B sim C 3671 T	Merimbula, NSW, AUS	14/12/1996
B sim C 3663 MF	Bermagui, NSW, AUS	13/12/1996
B sim C 3663 G	Bermagui, NSW, AUS	13/12/1996
B sim C 3663 F	Bermagui, NSW, AUS	13/12/1996
B sim C 3663 T	Bermagui, NSW, AUS	13/12/1996
B sim C 3581 T	Williamstown, VIC, AUS	29/03/1996
B sim C 3581 S	Williamstown, VIC, AUS	29/03/1996
B sim C 3581 F	Williamstown, VIC, AUS	29/03/1996
B sim C 3304 T	Merimbula, NSW, AUS	01/12/1993
B sim C 3108 T	Williamstown, VIC, AUS	30/12/1990
B sim C 3108 M	Williamstown, VIC, AUS	30/12/1990
B sim C 3108 F	Williamstown, VIC, AUS	30/12/1990
B sim C 3658 T	Sussex Inlet, NSW, AUS	14/12/1996
B sim C 3658 G	Sussex Inlet, NSW, AUS	14/12/1996
B sim C 3658 F	Sussex Inlet, NSW, AUS	14/12/1996
B sim C 3576	Woolooware Bay, NSW, AUS	14/12/1995
B sim C 3319 T	Narooma, NSW, AUS	01/12/1993
B sim C 2915 G	Torrens Island, SA, AUS	22/09/1988
B sim C 2915 T	Torrens Island, SA, AUS	22/09/1988
B sim C 2915 S	Torrens Island, SA, AUS	22/09/1988
B sim C 2747	Tooradin, Western Port Bay, VIC, AUS	25/10/1986
B sim C 3315 T	Tuross Lake, NSW, AUS	01/12/1993
B sim C 3672	Sussex Inlet, NSW, AUS	14/12/1996
B sim C 3657 T	Sussex Inlet, NSW, AUS	14/12/1996
B sim C 3612 T	Williamstown, VIC, AUS	21/05/1996
B sim C 3612 B	Williamstown, VIC, AUS	21/05/1996
B sim C 3612 F	Williamstown, VIC, AUS	21/05/1996
B sim C 3317 T	Narooma, NSW, AUS	01/12/1993
B sim C 4046	Between Pt. Clinton and Pt. Arthur, SA, AUS	01/10/2000
B sim C 2933	Millers Landing,Wilson's Promontory VIC, AUS	17/12/1988
B kin C 4203 F	Wooloware NSW, AUS	10/01/2001

Table S3. *Cont.*

B kin C 3545	Forster, NSW, AUS	22/10/1995
B kin C 3109 F	Coorumbine Ck, Gosford, NSW, AUS	01/04/1991
B kin C 3110 F	Coorumbine Ck, Gosford, NSW, AUS	01/04/1991
B kin C 3111 F	Coorumbine Ck, Gosford, NSW, AUS	01/04/1991
B kin C 3114 F	Coorumbine Ck, Gosford, NSW, AUS	01/04/1991
B kin C 3114 T	Coorumbine Ck, Gosford, NSW, AUS	01/04/1991
B kin C 3114 MT	Coorumbine Ck, Gosford, NSW, AUS	01/04/1991
B kin C 3308 TF	Broughton Ck., NSW, AUS	01/11/1993
B kin C 3308 F	Broughton Ck., NSW, AUS	01/11/1993
B sim C 2964 M	Lim Chu Kang, SGP	16/06/1989
B sim C 3211 T	AIMS, QLD, AUS	28/09/1991
B sim C 2963 B	Mandai, SGP	13/06/1989
B sim C 2963 T	Mandai, SGP	13/06/1989
B sim C 3992	Peleliu I., PLW	2/06/1999
B sim C 3546	Brunswick Heads, NSW, AUS	23/10/1995
B sim H Oo	Oomija River, Iriomote Island, Okinawa Japan	22/05/2019
B sim H Ka	Kanpire Falls in Urauchi River, Iriomote Island, Okinawa Japan	22/05/2019
B arb H	Wellington Noa Point	08/2016
B int C 2952	Bahia Ensenada, Tierra del Fuego, ARG	02/03/1989
B int C 2951 T	Caleta Cameron, Bahia Inutil, Tierra del Fuego, CHL	31/01/1989
B int C 4557	Umhlanga Rocks KwaZulu Natal ZAF	08/07/2005
B rad C 3029 F	San Carlos, Bahia Magdalena, B.C.S. MEX	01/07/1990
B rad C 3038 T	Bahia Balandra, B.C.S. MEX	01/06/1990
B rad C 3034 F	Bahia Balandra, B.C.S. MEX	01/06/1990
B rad C 3061 MF	Rio Sitio Grande, Ilha do Cardoso S.P. BRA	04/11/1990
B rad C 2928 F	Tampa, FL, USA	16/10/1988
B rad C 3129 MF	La Banqueta, Bahia Magdalena, B.C.S., MEX	25/03/1991
B rad C 3136 T	Soro, Edo Sucre, VEN	04/11/1991
B rad C 3124 MF	Estero Coyote, Bahia San Ignacio, B.C.S., MEX	26/03/1991
B rad C 2649	São Sebastião, São Paulo, BRA	07/02/1982
B rad C 2636	Itanhaem, São Paulo, BRA	14/06/1982
B rad C 3030 F	Bahia Balandra, B.C.S., MEX	01/06/1990
B rad C 3128 F	La Banqueta, Bahia Magdalena, B.C.S., MEX	25/03/1991
B rad C 3171 T	Isle of Palms, SC, USA	21/07/1991
B rad C 3137	Soro, Edo Sucre, VEN	04/11/1991
B rad C 3044 M	Puerto Pizarro, Tumbes, PER	02/10/1990
B rad C 3195 T	Charleston, SC, USA	10/09/1991

Table S3. *Cont.*

B rad C 3115 T	Cape Fear Estuary, NC, USA	27/01/1991
B rad C 3037 T	San Carlos, Bahia Magdalena, B.C.S., MEX	01/07/1990
B rad C 3026 MF	San Carlos, Bahia Magdalena, B.C.S. MEX	01/07/1990
B rad C 3062 T	Rio Sitio Grande, Ilha do Cardoso S.P. BRA	04/11/1990
B rad C 3035 F	Bahia Balandra, B.C.S. MEX	01/06/1990
B rad C 3051 T	Rio Pereque, Ilha do Cardoso, S.P., BRA	04/04/1990
B rad C 3058 MF	Rio Sitio Grande, Ilha do Cardoso S.P. BRA	04/11/1990
B rad C 3144 MF	Punta Uricaro, Edo Sucre, VEN	04/10/1991
B rad C 3125 MF	Puerto Escondido, B.C.S., MEX	23/03/1991
B rad C 3094 F	Sapelo Island, GA, USA	15/12/1990
B rad C 3203 V	St. Lucia, Natal, ZFA	10/04/1991
B rad C 3142 T	Punta Piedra, Bahia Mochima, Edo Sucre, VEN	04/09/1991
B rad C 3126 F	El Requeson, Bahia Concepcion, Baja California Sur, MEX	23/03/1991
B rad C 3140 M	Soro, Edo Sucre, VEN	04/11/1991
B rad C 3019 F	Mulege, B.C.S., MEX	01/08/1990
B tan C 3242 MFT	St. Lucia, Natal, ZFA	22/12/1991
B tan 3241 C MFT	St. Lucia, Natal, ZFA	20/01/1992
B tan C 3240 MFT	St. Lucia, Natal, ZFA	22/12/1991
B tan C 3239 MFT	St. Lucia, Natal, ZFA	22/12/1991
B tan C 3238 MFT	Durban, Natal, ZFA	15/11/1991
B ano C 4613 TMF	Kosrae, FSM	02/08/2006
B ano C 4588 V	Achang Bay Resort, GUM	02/12/2006
B vag C 3781	Millers Landing, Wilsons Promontory, VIC, AUS	10/11/1997
B har C 3908 F	Tarwin R., VIC AUS	12/07/1998
B ten H 4311 T	Angeva, MDG	22/04/2003
B ten C 2985	Batam I., IDN	17/06/1989
B ten H 2985 F	Batam I., IDN	17/06/1989
B ten C 3706	Barrow I., WA, AUS	02/08/1997
B ten C 4528 V	Eton Beach, Efate Island, VUT	14/06/2005
B ten C 2765 T	Siayan I., Batanes, PHL	31/10/1986
B ten H 2765 T	Siayan I., Batanes, PHL	31/10/1986
B ten C 2515	La Parguera, PRI	20/03/1981
B ten H 2515	La Parguera, PRI	20/03/1981
B ten C 4552 V	Umhlanga Rocks KwaZulu Natal ZAF	17/08/2005
B ten H 4552 V	Umhlanga Rocks KwaZulu Natal ZAF	17/08/2005
B ten C 2722	Deguey Is., PHL	05/10/1986
B ten H 2722 TF	Deguey Is., PHL	05/10/1986

Table S3. *Cont.*

B ten C 2850	Florence Bay, Magnetic I., QLD, AUS	06/04/1987
B ten H 2850 T	Florence Bay, Magnetic I., QLD, AUS	4/06/1987
B ten C 4229 M	Pajinka, Cape York, QLD, AUS	19/06/2002
B ten H 4229 M	Pajinka, Cape York, QLD, AUS	19/06/2002
B ten C 4089 B	Pulai Bai, Sandakan, Sabah, Malaysia,	16/08/2000
B ten H 4089 B	Pulai Bai, Sandakan, Sabah, Malaysia,	16/08/2000
B ten C 3727	Koro Levu, Viti Levu, FJI	06/04/1997
B ten H 3727 M	Koro Levu, Viti Levu, FJI	06/04/1997
B ten C 2998 T	Galeta, PAN	20/08/1989
B ten H 2998 T	Galeta, PAN	20/08/1989
B ten C 2908	Libertad, Initao, Misamis Oriental, PHL	06/09/1988
B ten H 2908	Libertad, Initao, Misamis Oriental, PHL	06/09/1988
B ten C 2905	Initao, Misamis Oriental, PHL	14/03/1988
B ten H 2905 T	Initao, Misamis Oriental, PHL	14/03/1988
B ten C 2815	Bouncing Stones Beach, QLD, AUS	13 /06/1987
B ten H 2815 T	Bouncing Stones Beach, QLD, AUS	13 /06/1987
B ten C 4662	Peniyak Village, Weno I., Chuuk, FSM	02/11/2006
B ten H 4662 V	Peniyak Village, Weno I., Chuuk, FSM	02/11/2006
B ten C 3807	Port Hedland, WA, AU	12/09/1997
B ten H 3807	Port Hedland, WA, AU	12/09/1997
B ten C 3079	Talabong, Negros Oriental, PHI	19/07/1990
B ten H 3079	Talabong, Negros Oriental, PHI	19/07/1990
B ten C 2767	Y'Ami I., Batanes, PHL	33/10/1986
B ten H 2767 F	Y'Ami I., Batanes, PHL	33/10/1986
B ten C 4116 F	Rookery Bay, FL, USA	19/09/2000
B ten H 4116 F	Rookery Bay, FL, USA	19/09/2000
B ten C 2979 T	Twin Cays, BLZ	15/07/1989
B ten H 2979 T	Twin Cays, BLZ	15/07/1989
B ten C 2978	Twin Cays, BLZ	15/07/1989
B ten H 2978	Twin Cays, BLZ	15/07/1989
B ten C 2764	Initao, Misamis Oriental, PHL	30/10/1986
B ten H 2764 F	Initao, Misamis Oriental, PHL	30/10/1986
B ten C 2904	Talisoy, Virac, Catanduanes, PHL	14/05/1988
B ten H 2904 T	Talisoy, Virac, Catanduanes, PHL	14/05/1988
B ten C 4081 F	Sikuati beach, Sabah, MYS	13/08/2000
B ten H 4081 F	Sikuati beach, Sabah, MYS	13/08/2000
B ten C 4081 M	Sikuati beach, Sabah, MYS	13/08/2000
B ten H 4081 M	Sikuati beach, Sabah, MYS	13/08/2000

Table S3. *Cont.*

B ten C 3417	Miami R., Miami FL, USA	14/06/1994
B ten H 3417 F	Miami R., Miami FL, USA	14/06/1994
B ten C 4434 V	River Sangara, Belo sur Mer MDG	27/05/2004
B ten H 4434 V	River Sangara, Belo sur Mer MDG	27/05/2004
B ten C 2751	Initao, Misamis Oriental, PHL	25/10/1986
B ten H 2751 F	Initao, Misamis Oriental, PHL	25/10/1986
B ten C 4096 F	Kuala Tanjungbatu, Sandakan, Sabah, MYS	16/08/2000
B ten H 4096 F	Kuala Tanjungbatu, Sandakan, Sabah, MYS	16/08/2000
B ten C 3635	Korotoga Village, Viti Levu, FIJ	06/09/1997
B ten H 3635 T	Korotoga Village, Viti Levu, FIJ	06/09/1997
B mor C 4170	Blue Lagoon Shriimp Farm Plage de Foué NCL	07/02/2001
B mor C 4169	Blue Lagoon Shriimp Farm Plage de Foué NCL	07/02/2001
B mor C 4168	Blue Lagoon Shriimp Farm Plage de Foué NCL	07/02/2001
B mor C 4167	Poé Beach, NCL	07/04/2001
B mor C 3905 M	Foster Beach, VIC AUS	12/07/1998
B mor C 3799	Mangrove Bay, WA, AUS	12/10/1997
B mor C 3700	Jacobs Well, QLD, AUS	13/02/1997
B mor C 3699	Tweeds Heads South, NSW, AUS	02/12/1997
B mor C 3659 T	Minnamurra River, NSW, AUS	14/12/1996
B mor C 4383 T	Port Douglas, QLD AUS	10/12/2003
B mor C 4371 T	near Ponerihouen. NCL	09/08/2003
B mor C 4369 T	Voh, Plage de Gatope, NCL	09/06/2003
B mor C 4164	Nera R. mouth, near Bourail NCL	07/04/2001
B mor C 4366 FT	Voh, Plage de Gatope, NCL	09/06/2003
B mor C 4309 T	Angeva, MDG	22/04/2003
B mor C 4310 T	Angeva, MDG	22/04/2003
B mor C 4278 V	Pichavaram , Tamil Nadu, IND	10/12/2002
B mor C 4163	Kouaoua NCL	07/03/2001
B mor C 4160	Kouaoua NCL	07/03/2001
B mor C 4631	Peniyak Village, Weno I., Chuuk, FSM	02/11/2006
B mor C 4363 V	Cape Bocage, N. of Houailou, NCL	09/08/2003
B mor C 4367 T	Hienghene, NCL	09/08/2003
B mor C 4609 T	Kosrae, FSM	02/07/2006
B mor C 4592 T	Nett Point, Pohnpei, FSM	02/05/2006
B mor C 4591 T	Nett Point, Pohnpei, FSM	02/05/2006
B mor C 4608	Kosrae, FSM	02/07/2006
B mor C 4633	Peniyak Village, Weno I., Chuuk, FSM	02/11/2006
B mor C 4590 V	Nett Point, Pohnpei, FSM	02/05/2006

Table S3. *Cont.*

B mor C 3001 T	Dehetik Island, Pohnpei, FSM	29/08/1989
B mor C 4596 F	Nett Point, Pohnpei, FSM	02/05/2006
B mor C 4368 T	before Poum, NCL	09/06/2003
B fla C 4400 T	Annan R., QLD AUS	10/09/2003
B fla H 4400	Annan R., QLD AUS	10/09/2003
B fla C 3553 T	Rainbow, QLD, AUS, coll. Ulf Karsten	29/10/1995
B fla H 3553	Rainbow, QLD, AUS, coll. Ulf Karsten	29/10/1995
B fla H 3821 T	Plage de Ouano, NCL	02/01/1998
B fla C 3821 T	Plage de Ouano, NCL	02/01/1998
B fla C 3884 T	St Lawrence, QLD, AUS	25/07/1998
B fla H 3884 T	St Lawrence, QLD, AUS	25/07/1998
B fla C 4218 T	Krabi, THA	17/03/2002
B fla H 4218 T	Krabi, THA	17/03/2002
B fla C 4230	Punsand Bay Cape York, QLD, AUS	19/06/2002
B fla H 4230	Punsand Bay Cape York, QLD, AUS	19/06/2002
B fla H 3113	Coorumbine Ck, Gosford, NSW, AUS	01/04/1991
B cal C 4097	Kuala Tanjungbatu, Sandakan, Sabah, MYS	16/08/2000
B cal C 4206	Krabi, THA	17/03/2002
B cal C 3252	San Blas, Nayarit, MEX	22/03/1992
B cal C 3190 T	Buenaventura, COL	09/10/1991
B cal C 3066 T	Rio Sitio Grande, Ilha do Cardoso S.P. BRA	04/05/1990
B cal C 3194 MF	Buenaventura, COL	09/10/1991
B cal C 3042	Rio Sitio Grande, Ilha do Cardoso S.P. BRA	18/01/1990
B cal C 3041 T	Rio Sitio Grande, Ilha do Cardoso S.P. BRA	18/01/1990
B cal C 3880	bridge crossing Calliope R, Gladstone, QLD, AUS	25/07/1998
B cal C 3414	Intracoastal Waterway, Ft. Pierce, FL, USA	18/06/1994
B cal C 3054 T	Rio Pereque, Ilha do Cardoso, S.P., BRA	04/05/1990
B cal C 3016	Puerto Pizarro, Tumbes, PER	02/10/1990
B cal C 3216 M	Chunda Bay, QLD, AUS	28/09/1991
B cal C 4194	Timika, Irian Jaya, IDN	29/12/2001
B rdco C 4271	Mooball Creek, NSW AUS	28/09/2002
B rdco C 4178	Plage de Foué Fishing Village NCL	07/02/2001
B rdco C 4086	Tempusak (near Kota Belud), Sabah, MYS	13/08/2000
B rdco C 3744	Streeters Jetty, Broome, WA, AUS	18/06/1997

Supplementary Materials References

1. West, J.; Pueschel, C.M.; Klochkova, T.A.; Kim, G.H.; De Goer, S.; Zuccarello, G.C. Gall structure and specificity in *Bostrychia* culture isolates (Rhodomelaceae, Rhodophyta). *ALGAE* **2013**, *28*, 83–92, doi:10.4490/algae.2013.28.1.083.
2. Zuccarello, G.C.; West, J.; Kamiya, M. Non-monophyly of *Bostrychia simpliciuscula* (Ceramiales, Rhodophyta): Multiple species with very similar morphologies, a revised taxonomy of cryptic species. *Phycol. Res.* **2017**, *66*, 100–107, doi:10.1111/pre.12207.
3. Zuccarello, G.C.; West, J.A.; King, R.J. Evolutionary divergence in the *Bostrychia moritziana*/B. radicans complex (Rhodomelaceae, Rhodophyta): molecular and hybridization data. *Phycol.* **1999**, *38*, 234–244, doi:10.2216/i0031-8884-38-3-234.1.
4. West, J.A.; Kamiya, M.; De Goer, S.L.; Karsten, U.; Zuccarello, G.C. Observations on some mangrove-associated algae from the western Pacific (Guam, Chuuk, Kosrae, and Pohnpei). *ALGAE* **2013**, *28*, 241–266, doi:10.4490/algae.2013.28.3.241.
5. West, J.A.; De Goer, S.L.; Zuccarello, G.C. Monosiphonous growth and cell-death in an unusual Bostrychia (Rhodomelaceae, Rhodophyta): B. anomala sp. nov. *ALGAE* **2013**, *28*, 161–171, doi:10.4490/algae.2013.28.2.161.
6. Zuccarello, G.C.; West, J.A. MULTIPLE CRYPTIC SPECIES: MOLECULAR DIVERSITY AND REPRODUCTIVE ISOLATION IN THE BOSTRYCHIA RADICANS/B. MORITZIANA COMPLEX (RHODOMELACEAE, RHODOPHYTA) WITH FOCUS ON NORTH AMERICAN ISOLATES. *J. Phycol.* **2003**, *39*, 948–959, doi:10.1046/j.1529-8817.2003.02171.x.
7. Kim, G.H.; Shim, J.B.; Klochkova, T.A.; West, J.A.; Zuccarello, G.C. THE UTILITY OF PROTEOMICS IN ALGAL TAXONOMY:BOSTRYCHIA RADICANS/B. MORITZIANA(RHODOMELACEAE, RHODOPHYTA) AS A MODEL STUDY1. *J. Phycol.* **2008**, *44*, 1519–1528, doi:10.1111/j.1529-8817.2008.00592.x.
8. Orfanoudaki, M.; Hartmann, A.; Karsten, U.; Ganzera, M. Chemical profiling of mycosporine-like amino acids in twenty-three red algal species. *J. Phycol.* **2019**, *55*, 393–403, doi:10.1111/jpy.12827.
9. Muangmai, N.; West, J.A.; Zuccarello, G.C. Evolution of four Southern Hemisphere Bostrychia (Rhodomelaceae, Rhodophyta) species: phylogeny, species delimitation and divergence times. *Phycol.* **2014**, *53*, 593–601, doi:10.2216/14-044.1.
10. Karsten, U.; Sawall, T.; West, J.; Wiencke, C. Ultraviolet sunscreen compounds in epiphytic red algae from mangroves. *Hydrobiol.* **2000**, *432*, 159–171, doi:10.1023/A:1004046909810.
11. Zuccarello, G.C.; West, J. Bostrychia (Rhodomelaceae, Rhodophyta) species of New Zealand, and relationships in the Southern Hemisphere. *New Zealand J. Mar. Freshw. Res.* **2008**, *42*, 315–324, doi:10.1080/00288330809509959.
12. West, J.; Klochkova, T.A.; Kim, G.H.; Goër, S.L.-D. Olpidiopsis sp., an oomycete from Madagascar that infects Bostrychia and other red algae: Host species susceptibility. *Phycol. Res.* **2006**, *54*, 72–85, doi:10.1111/j.1440-1835.2006.00410.x.
13. Zuccarello, G.C.; Muangmai, N.; Preuss, M.; Sanchez, L.B.; De Goer, S.L.; West, J.A. The Bostrychia tenella species complex: morphospecies and genetic cryptic species with resurrection of B. binderi. *Phycol.* **2015**, *54*, 261–270, doi:10.2216/15-005.1.
14. Zuccarello, G.C.; West, J.A. Molecular phylogeny of the subfamily Bostrychioideae (Ceramiales, Rhodophyta): subsuming Stictosiphonia and highlighting polyphyly in species of Bostrychia. *Phycol.* **2006**, *45*, 24–36, doi:10.2216/05-07.1.
15. Zuccarello, G.C.; West, J. Phylogeography of the Bostrychia calliptera–B. pinnata complex (Rhodomelaceae, Rhodophyta) and divergence rates based on nuclear, mitochondrial and plastid DNA markers. *Phycol.* **2002**, *41*, 49–60, doi:10.2216/i0031-8884-41-1-49.1.
